# Extracellular Vesicles from Red Blood Cells and Their Evolving Roles in Health, Coagulopathy and Therapy

**DOI:** 10.3390/ijms22010153

**Published:** 2020-12-25

**Authors:** Kiruphagaran Thangaraju, Sabari Nath Neerukonda, Upendra Katneni, Paul W. Buehler

**Affiliations:** 1 Center for Blood Oxygen Transport and Hemostasis, Department of Pediatrics, University of Maryland School of Medicine, Baltimore, MD 21201, USA; kthangaraju@som.umaryland.edu (K.T.); pbuehler@som.umaryland.edu (P.W.B.); 2 Department of Animal and Food Sciences, University of Delaware, Newark, DE 19716, USA; nnvsnath@udel.edu; 3 Center for Biologics Evaluation and Research, U.S. Food and Drug Administration, Silver Spring, MD 20993, USA; 4 Department of Pathology, University of Maryland School of Medicine, Baltimore, MD 21201, USA

**Keywords:** red blood cells, extracellular vesicles, exosomes, microvesicles, microparticles, cell-to-cell communication, homeostasis, coagulopathy

## Abstract

Red blood cells (RBCs) release extracellular vesicles (EVs) including both endosome-derived exosomes and plasma-membrane-derived microvesicles (MVs). RBC-derived EVs (RBCEVs) are secreted during erythropoiesis, physiological cellular aging, disease conditions, and in response to environmental stressors. RBCEVs are enriched in various bioactive molecules that facilitate cell to cell communication and can act as markers of disease. RBCEVs contribute towards physiological adaptive responses to hypoxia as well as pathophysiological progression of diabetes and genetic non-malignant hematologic disease. Moreover, a considerable number of studies focus on the role of EVs from stored RBCs and have evaluated post transfusion consequences associated with their exposure. Interestingly, RBCEVs are important contributors toward coagulopathy in hematological disorders, thus representing a unique evolving area of study that can provide insights into molecular mechanisms that contribute toward dysregulated hemostasis associated with several disease conditions. Relevant work to this point provides a foundation on which to build further studies focused on unraveling the potential roles of RBCEVs in health and disease. In this review, we provide an analysis and summary of RBCEVs biogenesis, composition, and their biological function with a special emphasis on RBCEV pathophysiological contribution to coagulopathy. Further, we consider potential therapeutic applications of RBCEVs.

## 1. Introduction

Extracellular vesicles (EVs) are a heterogenous population of membrane-delimited organelles released into the extracellular milieu by eukaryotic and prokaryotic cells [1]. The first EV-like particles were suggested by Edward G. Horder in the late 1800s [2]. In 1967, Peter Wolf identified vesicles derived from activated platelets and termed them “platelet dust” [3,4]. The first description of erythropoietic cell origin EVs was provided by Johnstone et al. in 1987 using in vitro cultured sheep reticulocytes [4]. EVs were reported to be present in blood and other body fluids including saliva, breast milk, urine, semen, sputum, cerebrospinal fluid, and nasal fluid [5,6,7]. Under both physiological and pathological conditions, various cell types including endothelial cells, lymphocytes, dendritic cells, mast cells, platelets, leukocytes, erythrocytes, cancer cells, hematopoietic cells, neurons, and oligodendrocytes were reported to secrete EVs [1,8,9].

Originally thought of as a means to dispose of cellular waste products, EVs are now regarded as vehicles of intercellular communication that impact several physiological and pathological processes [8,9]. Despite their heterogeneity, EVs overlap in size, density, and content, which poses a significant technical challenge for separation and characterization of distinct EV subpopulations [10]. Nevertheless, extensive characterization has led to a broad categorization of EVs on the basis of size (<200 nm (small EVs), or >200 nm (medium/large EVs)) or density (low, middle, high), biochemical composition (e.g., CD63+/CD81+-EVs, Annexin A5+ EVs, etc.) and cellular origin (e.g., oncosomes of tumor cells) or treatment condition (e.g., hypoxic EVs) [10]. Small EVs include exosomes whose size range falls between 50 and 150 nm in diameter whereas medium/large EVs comprise microvesicles and apoptotic bodies with size ranges <1 µM and 1–5 µm, respectively [10].

**Figure 1:**
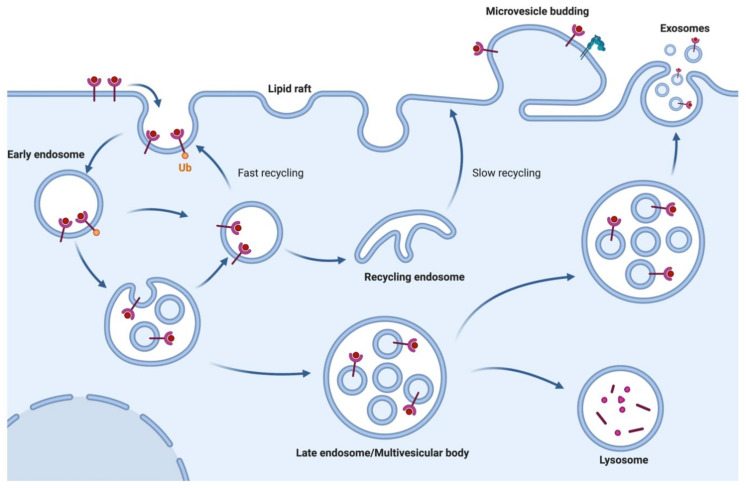
Biogenesis of extracellular vesicles (EVs; exosomes and microvesicles): EVs are broadly classified into two categories: exosomes and ectosomes or microvesicles. Exosomes demonstrate a size range between 40 to 150 nm and are generated through a process that involves double invagination of the endosomal membrane to form multivesicular bodies containing intraluminal vesicles. This process is followed by fusion of the multivesicular bodies to the plasma membrane to produce exosomes. By contrast, ectosomes (i.e., microvesicles) and large vesicles are generated by outward budding of the plasma membrane with a size range of 50 to 1000 nm in diameter. (Figure created with BioRender.com).

Exosomes are generated along the endosomal pathway after the first step of plasma membrane (PM) invagination to form early endosomes [11]. Early endosomes mature into late-sorting endosomes, which eventually become multivesicular bodies (MVBs) bearing intra-luminal vesicles (ILVs). ILVs are generated upon inward invagination of endosomal structures generating membrane-derived MVBs. Newly formed MVBs can either undergo targeted degradation after fusion with lysosomes and autophagosomes, or fuse with PM to release the ILVs as exosomes (Figure 1) [12,13,14,15,16]. The endosomal sorting complex required for transport (ESCRT) complexes primarily mediate inward budding of ILVs into MVBs and concomitantly the exosome cargo selection process [12,17]. Exosome release and uptake is a constitutive homeostatic process among many cell types except mature RBCs which lack the endocytic machinery [4,18]. Microvesicles and apoptotic bodies are generated upon PM exvagination and apoptotic blebbing, respectively (Figure 1) [19,20]. In the current review, small- and large-sized vesicles from RBCs are referred to exclusively as EVs since they are not yet fully characterized into independent categories.

## 2. EV Biogenesis

### 2.1. Exosome Biogenesis

In nucleated cells, EVs are defined either as exosomes or MVs and each have distinct subcellular origins. Exosome biogenesis was initially described in yeast where four ESCRT complexes (ESCRT-0, -I, -II and -III), along with their protein partners (e.g., ALIX, VPS34), sequentially perform cargo selection, inward membrane invagination, and scission, causing exosomes or ILV biogenesis [21]. ESCRT0 initially recruits ESCRTI complex in order to cluster ubiquitinated protein cargo on endosomal membrane microdomains. Upon complexing with ESCRTII subunits, both ESCRT-I and –II complexes initiate the invagination, while ESCRTII recruits the ESCRTIII complex into the neck of nascent ILVs to mediate budding and vesicle scission [22,23,24,25,26]. An additional pathway of ILV biogenesis that occurs in an ESCRTIII subunit (CHMP4) dependent manner is mediated by syndecan-syntenin-ALIX complex [27].

Alternatively, exosomes can also be formed in an ESCRT-independent manner [28,29,30]. Exosome formation is still evident in cells depleted of all four ESCRT complexes [28]. ESCRT-independent mechanisms of exosome biogenesis involve generation of membrane subdomains through the ceramide or tetraspanin protein family [29,30,31]. Neutral type II sphingomyelinase hydrolyses sphingomyelin to ceramide, which in turn coalesces smaller membrane microdomains into larger domains that drive domain-induced ILV budding [30]. However, ceramide is sequentially metabolized into sphingosine 1-phosphate (S1P), which permits cargo sorting into ILVs by continuous activation of inhibitory G (G_i_)-protein-coupled S1P receptor [32]. The tetraspanin family of proteins (CD9, CD63, CD81, and CD82) generate budding microdomains by forming clusters and membrane platforms between each other and with various other transmembrane and cytosolic proteins [29,31]. Among tetraspanins, CD63 is a bona fide exosomal marker that was demonstrated to be involved in endosomal sorting, ILV cargo sorting, and exosome biogenesis in melanocytes [13,29], melanoma cells [33], and fibroblasts [34], respectively. Furthermore, tetraspanins are also involved in routing cargo, such as integrins to multivesicular endosomes, indicating their role in cargo sorting apart from exosome biogenesis [35].

Exosomes are packaged in endosomes during the process of erythropoiesis from hematopoietic stem cells through erythropoietin-dependent cellular maturation ending in erythroblasts formation. By the time iron dependent hemoglobin (Hb) synthesis begins in the process of reticulocyte maturation to erythrocytes, exosomes are defined and packaged [36,37,38]. Circulating reticulocytes and erythroid precursors contain components of ESCRT complexes required for exosome biogenesis. Proteomic analysis of exosomes derived from human cord blood reticulocytes as well as exosomes derived from human and murine reticulocytes has identified ESCRT proteins involved in MVB biogenesis. These proteins—including Hrs (ESCRT-0), TSG101 (ESCRT-I), Alix (ESCRT-II), and CHMP4B (ESCRT-III)—highlight identical mechanisms of exosome biogenesis as described above [39,40]. These particular ESCRT proteins were also identified in the human RBC proteome; however, their RBC specific roles in the process of vesiculation remains to be determined [41] and there remains a possibility that they simply represent carry over of EVs derived from nucleated cells.

### 2.2. MV Biogenesis

**Figure 2:**
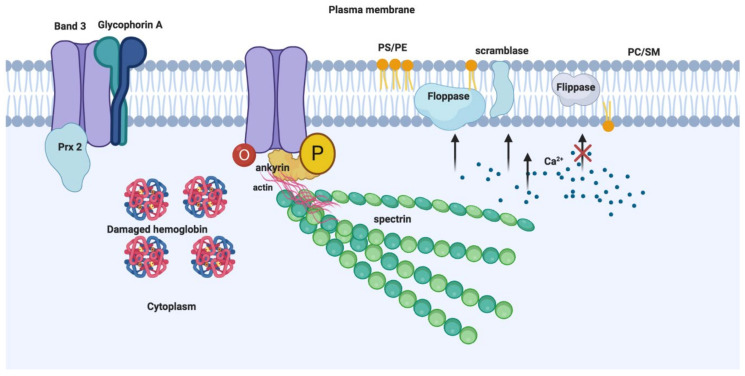
Mechanisms of RBC-MV biogenesis: MV generation on RBC membrane is predominantly triggered by damaged hemoglobin, protein oxidation, and senescence-associated degradation of band 3-cytoskeletal ankyrin association which result in evagination and vesiculation. Another mechanism involves the alterations in phospholipid distribution in lipid bilayer. Enzymes such as scramblase, calpain, and proteases are activated by oxidative damage or Ca^2+^ influx via nonspecific cation channels leading to inhibition of flippase and phosphatidylserine externalization, cytoskeletal proteolytic degradation, and band 3 aggregation, resulting in vesiculation. Peroxiredoxin 2 (Prx-2) binding to N-terminal cytoplasmic domain of band 3, phosphorylation (P) and oxidation (O) of band 3 are also indicated [67]. (Figure created with BioRender.com).

MV biogenesis and release is an integral part of RBC physiology that is coupled with RBC maturation and ageing that facilitates the timely disposal of damaged RBC components, which otherwise may trigger unwanted hemostatic and immunological reactions [42]. Microvesicle release during RBC maturation is a mechanism to dispose of unwanted proteins, and to alter the cell volume to surface area to enable membrane remodeling [37]. MVs containing acetylcholinesterase, membrane proteins (e.g., transferrin receptor), glucose membrane transporters (e.g., GLUT-1), and amino acid transporters (e.g., excitatory amino acid transport, EAAT family) might contribute to RBC membrane remodeling during maturation [37]. MV shedding by ageing RBCs contributes to the loss of Hb (approximately 20%) and cell membrane with a concomitant decrease in cell volume and an increase in cell density [43,44,45]. The Hb composition of RBC MVs is enriched with irreversibly modified species (HbA1c and HbA1e2) that are typically identified in senescent RBCs in dense fractions [44,46]. RBC MV biogenesis involves profound alterations in the anatomy of RBC membrane that is made of phospholipids and several integral membrane proteins present as macromolecular complexes centered on the anion-exchange channel, band 3 [47,48]. The membrane cytoskeleton laminating the inner membrane surface comprises cytoskeletal proteins, spectrin [49,50], actin [51] and its associated proteins (tropomyosin, tropomodulin, adducin, and dematin) [52,53,54,55], protein 4.1R [56,57], and ankyrin [47]. Band 3 macromolecular complexes are dynamic with bound and unbound integral membrane proteins (e.g., CD44, CD47, glycophorins) or peripheral membrane proteins (e.g., glycolytic and redox enzymes) [41] (Figure 2). Proteomic and immunoblot analyses of RBC MVs from the blood plasma of healthy individuals indicate identification of membrane or cytoskeletal proteins, aggregated specifically with band 3 and actin, absent spectrin and ankyrin [46]. Furthermore, RBC MVs contain elevated concentrations of enzymes involved in redox homeostasis, including glutathione S transferase, thioredoxin, and peroxiredoxin-1 and -2 in vesicles compared to erythrocyte membranes [46]. In addition, the MV membrane contains removal signals such as phosphatidylserine (PS) and immunoglobulins (Igs) [46], whereas RBC membrane is highly enriched in proteasome subunits and ubiquitin [46]. These contents highlight several putative upstream processes, including, but not limited to, Hb damage, protein oxidation, and senescence-associated degradation of band 3-cytoskeletal ankyrin association as predominant triggers for MV generation [46]. Dismantling band 3-ankyrin binding eventually relieves the connection between the cytoskeleton and the lipid bilayer, resulting in exvagination and vesiculation [42,58]. These processes are evident in hemoglobinopathies including sickle cell anemia (SCA) and thalassemia intermedia (TI) where increased levels of circulatory MVs correlate with plasma Hb concentrations and accumulation of degraded Hb [59]. Similarly, in TI patients, α or β oxidation form unstable membrane-bound hemichromes, which facilitate band 3 oxidation by the release of free iron radicals [60]. Oxidized band 3 dimers are subject to phosphorylation by p72Syk kinase on Tyr 8 and 21 residues of band 3 cytoplasmic domain to result in the weakening of its association with cytoskeleton and greater lateral mobility causing subsequent aggregation [60]. Aggregated band 3 and hemichromes are released into MVs. Aggregated band 3 is also bound by anti-band 3 Igs that subsequently undergo phagocytic removal. Consistent with this process, proteomic analyses of TI MVs identified denatured α-globin, Igs and a number of proteins involved in redox homeostasis (e.g., HSP90, HSP70, catalase, and peroxiredoxin-2) [60]. The presence of PS, C3, and Igs facilitate the removal of MVs by liver Kupffer cells and spleen red pulp macrophages [61]. The presence of glycosylphosphatidylinositol (GPI)-anchored complement-inhibiting proteins CD55 and CD59 on MV surfaces may prevent unwanted activation of complement during vesicle removal, if functionally configured [42].

Alterations in phospholipid distribution in the lipid bilayer suggest another mechanism of RBC MV biogenesis. RBC membrane integrity is maintained by four major classes of phospholipids that are asymmetrically segregated on opposite leaflets of the bilayer (Figure 2). The choline-based phospholipids, sphingomyelin (SM), and phosphatidylcholine (PC) are enriched in the outer leaflet, whereas the primary amine-based phospholipids, phosphatidylethanolamine (PE), and phosphatidylserine (PS) are enriched on the inner cytoplasmic leaflet with PS displaying an absolute distribution [62]. This lipid asymmetry is maintained by phospholipid transporter enzymes known as flippases (inward), floppases (outward), and scramblases (bidirectional) [63]. Under resting conditions, when the cytoplasmic Ca^2+^ concentration is low, flippases internalize negatively charged PS whereas floppase and scramblase remain inactive. Activation or inactivation of phospholipid transporter enzymes by triggers causing oxidation or Ca^2+^ influx was known to induce vesiculation [64,65,66] (Figure 2).

Ca^2+^ influx via nonspecific cation channels promotes the activation of calpain protease and scramblase and inhibits flippase leading to PS externalization, cytoskeletal proteolytic degradation and band 3 aggregation, all of which promote RBC membrane vesiculation [63,68]. Accordingly, incubation of RBCs with Ca^2+^ ionophore A23187, lysophosphatidic acid (LPA) or phorbol-12-myristate-13-acetate (PMA) to elevate intracellular Ca^2+^ concentration results in PS externalization, promoting the release of MVs, while incubation with a scramblase specific inhibitor (R5421) to prevent PS externalization significantly limits the release of MVs [65]. Notably, Ca^2+^ and A23187 incubation with erythrocytes also promotes co-release of nanovesicles (NVs), sized 60nm in diameter [69], whereas MVs are specifically enriched for lipid raft protein stomatin, raft proteins, synexin, and sorcin were observed in both MVs and NVs. Therefore, Ca^2+^ dependent vesicle release is a raft-based process. These studies do stress an important role for Ca^2+^ in the liberation of MVs or NVs from RBCs; however, non Ca^2+^ dependent pathways are also observed [65,70,71]. Specifically, limiting oxidative stress by the addition of small molecule antioxidants (e.g., ascorbic acid) during blood banking conditions reduces MV release over the time course of storage and also attenuates alloimmunogenicity in murine models of transfusion [72].

Different from RBCs, several mechanisms of MV biogenesis are defined in nucleated cells. Arrestin-domain-containing protein 1 (ARRDC1) -mediated microvesicles (ARMMs) of ~50 nm diameter was demonstrated to bud directly from the PM, similar to virus budding at PM, in a manner that is dependent on ARRDC1 nucleation and ARRDC1-Tsg101 interaction at PM [73]. In cardiomyocytes, ampiphysin-1 (Bin-1) organized membrane microdomains promote actin polymerization and MV release in a CHMP4B-dependent manner [74]. In breast (MDA-MB231) and cervical carcinoma (HeLa) cell lines, activation of RhoA/ROCK signaling and sequential downstream phosphorylation of LIMK1 and cofilin was demonstrated to induce MV budding [75,76]. In monocytes, cholesterol bearing lipid rafts are required to produce MVs [77]. In enterocytes, microvillar tips generate unilamellar vesicles into the lumen in myosin-1a dependent manner [78]. Except for the role of lipid raft-based processes, relevance of the remaining mechanisms in RBCEV biogenesis is unknown and none of the major protein regulators of EV biogenesis were identified in either the RBC or RBCEV proteome. Therefore, RBCEV generation represents distinct and cell specific biogenesis mechanisms.

## 3. Molecular Composition of RBCEVs

**Figure 3:**
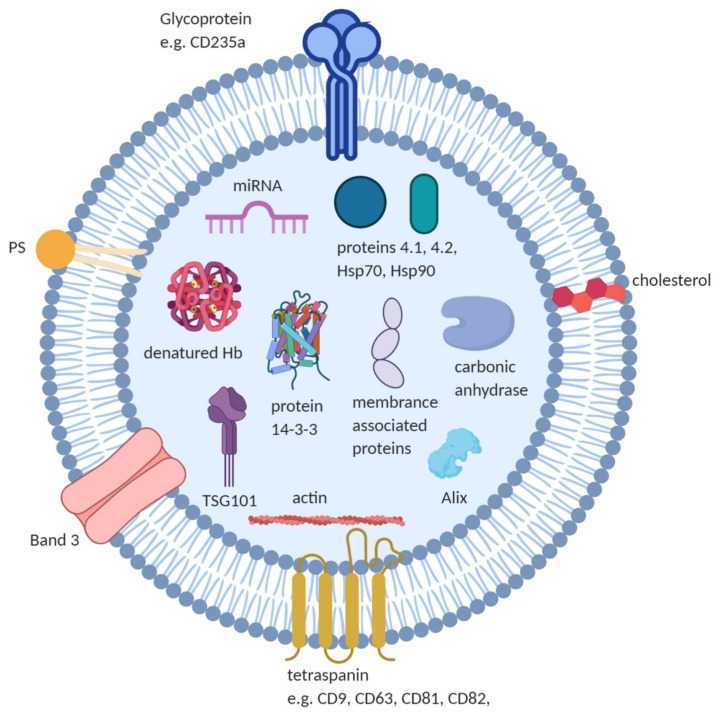
Composition of RBCEVs: RBCEVs are reported to contain cytoskeletal proteins (e.g., actin), irreversibly modified Hb, anion transport proteins (e.g., Band 3), glycoproteins (e.g., CD235a), proteins 4.1, 4.2, and 14-3-3, multivesicular body fusion proteins (e.g., Alix, TSG101), membrane-associated proteins (e.g., stomatin (Band 7.2b) and flotillin) and enzymes like carbonic anhydrase. Negatively charged phospholipids (e.g., phosphatidylserine) and other lipid molecules such as cholesterol, and nucleic acid such as miRNA are reported in RBCEVs. (Figure created with BioRender.com).

Proteomic and transcriptomic studies have characterized the protein and nucleic acid components of the RBCEVs. Some of the contents of RBCEVs are shown in Figure 3 and selected studies listing composition, techniques and key findings are listed in the Supplementary Table S1. RBCEVs Information gathered from these studies have been routinely deposited in databases like EVpedia, Exocarta, and Vesiclepedia [79,80,81] and ExoCarta [82].

The proteomic profiles of total membrane protein extracts and band 3 complexes from stored RBCEVs are described [83]. Comparison of membrane protein extract from RBCs and EVs revealed 32 common proteins, 26 RBC-specific and 25 EV-specific proteins with an enrichment of acetylcholinesterase in EVs. RBCs and EVs from band 3 complex preparations had 11 common proteins, 5 RBC-specific and 7 MV-specific proteins. Compared to RBCs, band 3 complex derived EVs lacked spectrins but exclusively contained complement C4 and galectin 7. In the membrane protein preparations, EVs were devoid of membrane-skeleton linking proteins such as ankyrin, proteins 4.1 and 4.2. EVs generated during blood banking are enriched in glycophorin A, lipid raft proteins stomatin and synexin, but depleted in actin compared to intact RBCs [63,84,85]. RBC oxidation increases within 3 weeks of storage leading to significantly higher levels of carbonylated proteins in vesicles [86].

MicroRNAs (miRNAs) are a class of small RNAs (20–30nt in length) enriched in RBCs that control gene expression via target mRNA degradation or translation repression [87]. MicroRNAs are involved in regulation of cell differentiation and proliferation, development, apoptosis, hematopoiesis, tumorigenesis, and in different stages of erythropoiesis, including proliferation, differentiation, and maturation [88,89,90,91,92].

Approximately 78 miRNAs are reported within EVs isolated from stored RBCs (3 separate donor units) with a mean size of 64.08 nm. Three miRs—mir-125-b-5p, 4454, and 451a—were most abundant and present in all three donor unit isolated exosomes and miR-4454 and miR-451a levels were observed to increase with the duration of refrigerator storage time [93]. RNA sequencing [93] predicted target genes for the top ten most abundant RBC exosomal miRNAs: miR29a-3p, 101-3p, 125b-5p, 22-3p, 30b-5p, 451a, 30c-5p, 4454, 1260b, and 96-5P [93]. Among those identified, MiR-125b-5p acts as a negative regulator of inflammatory genes through the TRAF6/MAPKs/NF-κB pathway in human osteoarthritic chondrocytes [94] and modulates the inflammatory state of macrophages by targeting B7-H4 [95]. MiR-125b-5p mimics are reported to attenuate liver injury in murine models of acute liver failure [96] and act as anti-multiple myeloma agents in vitro and in vivo [97]. Doss et al. [98] reported 287 known and 72 putative novel miRNAs [98] with miR-451, 144, and 486, representing abundant genetic residual contents in mature erythrocytes [92]. MiRs-451 present in stored RBCEVs and miR-144 are located within the same gene cluster and are regulated by the erythroid transcription factor GATA1-binding factor 1 during erythropoiesis [92,99].

Furthermore, changes in the RBC microRNA profiles from low and high altitude populations [100] are described. MiRNA-144-5p and miR-30b-5p demonstrate increased expression levels in high altitude dweller’s RBCs based on RNA sequencing data and both miRNAs may be involved in erythroid and nitric oxide (NO) related signaling pathways during hypoxia. Some of the miRNAs reported in RBCEVs [93] were either upregulated (miR-30b-5p [101], miR-125b-5p [102], and miR-451a [102,103]) or downregulated (miR-101 [104]) during hypoxia. A comprehensive cataloging [105] of cell specific miRNAs derived from human peripheral blood identifies 271 RBC miRNAs, 90 serum miRNAs, and 5 miRNAs compartmentalized within exosomes. Some of these miRNAs are expressed in all the three portions, and some are unique to a particular component of the circulating blood [105].

## 4. Biological Roles of RBCEVs

**Figure 4:**
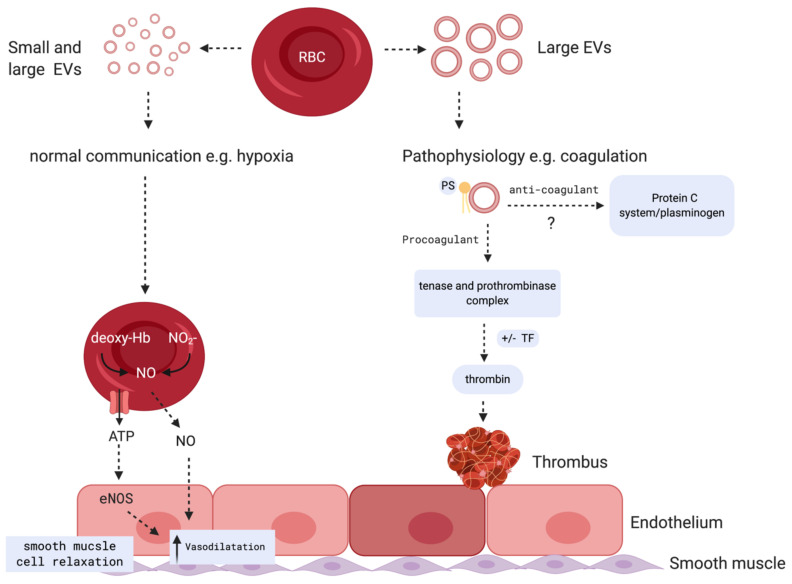
Potential biological role of RBCEVs: During hypoxia, small and large RBC vesicles carry factors that are responsible for NO production mediated by eNOS, resulting in an increase in vasodilation and smooth muscle cell relaxation (left panel). Large RBC vesicles are reported to play both pro and potential anti-coagulant roles (right panel): MVs mediate procoagulant activities by facilitating assembly of tenase and prothrombinase complexes on phosphatidylserine and promoting thrombin generation. Potential ability of MVs to mediate anticoagulant reactions through their interactions with protein S and activation of anticoagulant protein C system and plasminogen on their surface was reported. This process, in some circumstances, may create an anti-inflammatory and anti-coagulant response based on EV release from certain cells including neutrophils and platelets [116]. (Figure created with BioRender.com).

Under physiological and pathological conditions, RBCEVs loaded with proteins, lipids, and miRNAs might be vital for communication with the endothelium to regulate NO and O_2_ homeostasis, redox balance, and immunomodulation. Further, RBCEVs are critical to the dysregulation of hemostasis and demonstrate relevant pro-coagulant effects in several disease states. Roles of small and large RBCEVs during normal communication and pathophysiology are proposed in Figure 4.

### 4.1. Nitric Oxide Homeostasis

NO is an important signaling molecule that acts as a vasoregulator and modulates the vascular microenvironment. Oxygenated Hb becomes oxidized by NO through a deoxygenation reaction that generates metHb and nitrate [106]. Meanwhile, deoxygenated Hb binds NO with high affinity at the heme iron (Fe^2+^), altering [107] NO bioavailability and in turn affecting O2 homeostasis and vasoregulation. Studies suggest that EVs from packed red blood cell units scavenge NO at a slower rate than extracellular Hb, but at a faster than RBC-encapsulated Hb [108,109]. RBCEVs impact on NO bioavailability depends on several factors including the abundance of particles entering the microcirculation and their proximity to the endothelium. In vitro and in vivo studies have shown that RBCEVs by enhancing ROS production disturb NO homeostasis leading to endothelial dysfunction [110,111,112]. RBCEVs from JAK2^V617F^ myeloproliferative neoplasms increase endothelial oxidative stress leading to NO pathway inhibition [111].

### 4.2. Redox Balance

RBCs maintain a balance between the pro-oxidant and antioxidant status within the circulation. RBCs are well equipped with antioxidant enzymes such as thioredoxin reductase/peroxiredoxin system, superoxide dismutase, catalase, glutathione peroxidase, glutathione reductase, and reducing equivalents as well as non-enzymatic antioxidants: glutathione, ascorbic acid, a-tocopherol, and thioredoxin [113]. Further, RBCs function as critical compartments for the reduction of oxidized small molecule antioxidants such as dehydro-ascorbic acid. However, oxidative processes within the RBC that typically occur ex vivo (under storage conditions) or in vivo (following transfusion or during disease) leads to RBCEVs that can be involved in causing respiratory burst as well as neutrophil activation characterized by rapid release of the reactive oxygen species (ROS) [114,115]. Co-incubation of RBCEVs with neutrophils results in the generation of reactive oxygen species as well as transfer of vesicular components to cells. This effect of RBCEVs on neutrophils is suggested to be caused by the accumulation of lysophospholipid in vesicles that contribute toward the pathogenesis of transfusion-related acute lung injury [114].

### 4.3. Immunomodulation

The immunomodulatory effects of RBCEVs were reviewed in greater detail elsewhere [117,118,119]. In vitro studies suggest that mixing of RBCEVs with peripheral blood mononuclear cells (PBMCs) causes secretion of proinflammatory chemokines and cytokines and increases the survival of unstimulated PBMCs [115]. This induction of proinflammatory cytokines appears to be mediated by the interactions between the exosome fraction of either platelet, endothelial, or RBC origin EVs and monocytes [115]. Further RBCEVs amplified the replication of mitogen-induced CD4+ and CD8+ T-lymphocytes in an antigen-presenting cell dependent manner. Another study by Fisher et al. also demonstrated the induction of proinflammatory cytokines from PBMCs by RBCEVs. This study also demonstrated the increased interaction of platelets with neutrophils and monocytes upon incubation with RBCEVs. Additionally, RBCEVs from stored units bind to monocytes to activate endothelial cells by a β-integrin mediated process [120]. The interactions between RBCEVs and monocytes could impact post-transfusion complications by triggering proinflammatory cytokines secretion, and through neutrophil and platelets interactions [115,121].

After macrophage exposure to zymosan A and lipopolysaccharide, phagocytosed RBCEVs demonstrate immunosuppressive effects that lead to inhibition of tumor necrosis factor-α and release of interleukin 8 [122]. The immune suppressive effects of syngeneic RBC transfusion in murine models of delayed-type hypersensitivity are described [123]. Administration of syngeneic RBCs generate CD9 and CD81 positive EVs that are able to suppress delayed-type hypersensitivity mediated through miRNA-150 [123]. Further, a decrease in T-cell activation and an increase in apoptosis were observed when delayed-type hypersensitivity effector cells were treated with EVs from syngeneic RBCs in these models. EVs from *Plasmodium* infected RBCs exert their immunomodulatory role on human primary macrophages and neutrophils [116].

### 4.4. Critical Role for RBCEVs in Coagulopathy

The procoagulant activity of RBCEVs is well documented and represents the most well studied areas of RBCEV driven disease sequelae. The shortening of plasma clotting time by RBC lysates dates back to 1961 [124]. In 2006, experimental observations suggest that the addition of RBC lysate to intact RBC or platelets amplifies thrombin generation (TG) as evidenced by increased endogenous thrombin potential (ETP), maximal thrombin concentration and decreased time to reach peak TG [125]. This thrombogenic potential of RBC lysate was not observed when lysate was filtered through 0.22 μm filter. The data suggest an important role for RBC membranes and potentially RBCEVs rather than soluble proteins in the process of thrombogenesis. Phosphatidylserine (PS) exposed on the outer membrane is known to mediate the procoagulant activity of RBCEVs. The negatively charged PS interacts with gamma-carboxyglutamic acid (Gla) rich domains of coagulation factors in the presence of calcium acting as a docking site for the formation of tenase and prothrombinase complexes [126,127]. RBCEVs drive TG through the intrinsic pathway of coagulation because deficiency of factor XII, but not factor VII, is an inhibitor of TG. This observation also suggests a tissue factor independent initiation of coagulation [127]. Conversely, the ability of RBCEVs to interact with protein S and support activated protein C mediated anticoagulant reaction [128] and mediation of fibrinolytic activity, primarily from the presence of plasminogen on their surface [129] was also demonstrated. The significance of these anticoagulant interactions in disease states is not clear and to our knowledge not studied.

#### 4.4.1. Pro-Coagulant RBCEVs Generated under Blood Banking Conditions

RBCs stored ex-vivo under blood banking conditions intended for transfusion undergo several changes including loss of membrane and cell volume through shedding of RBCEVs [84,130]. A significant increase in the concentration of RBCEVs following storage at 4°C was reported by multiple studies [84,130,131]. Further, RBCEVs accumulated during refrigerated storage were demonstrated to express PS on their surface [130,132]. The procoagulant activity of RBCEVs secreted from stored RBCs is suggested by results that demonstrate significantly decreased clotting time, enhanced procoagulant activity [130], and increased TG [132,133]. Ex vivo storage of RBCs for transfusion may lead to the accumulation of cell-free Hb containing RBCEVs [108,109]. Hb containing RBCEVs act as scavengers of NO and lead to systemic vasoconstriction in rodent models of transfusion [108,109]. The ability of RBCEVs to scavenge NO is proposed to be dependent on their ability to reach the RBC-free layer, parallel to endothelial cells [109]. Under in vitro conditions, Hb containing RBCEVs were shown to transfer heme to human umbilical cord vascular endothelial cells and induced oxidative stress and apoptosis [134]. Further, loss of NO homeostasis activates platelets and promotes a pro thrombotic state [135,136]. It is suggested that the NO scavenging capability of Hb containing RBCEVs may contribute toward this process [137]. In murine models of SCA, injection of ex vivo generated Hb containing RBCEVs led to rapid vaso-occlusion within the renal glomerular circulation, while administration of the heme scavenger, hemopexin prevented renal vascular microthrombi [134]. Taken together, Hb containing RBCEVs can alter NO bioavailability and promote heme mediated endothelial dysfunction. Abnormal RBC metabolism is the primary driver of RBCEV accumulation, hemolysis, morphological changes, and reduced deformability that occurs during RBC refrigerator storage and each can individually or collectively contribute toward complications associated with transfusion [138].

#### 4.4.2. Pro-Coagulant RBCEVs Generated in Health and Disease

In healthy individuals, circulating EVs contribute to low grade TG. Depletion of microparticles from platelet-free plasma of healthy individuals results in delayed lag time and time to peak TG, as well as increased sensitivity to fibrinolysis [139,140,141]. However, differences in analysis of circulating EVs can generate differing results. For example, studies suggest no differences in the peak height value of TG or endogenous thrombin potential [141] of isolated circulation EVs, while other studies suggest a decrease in both parameters [139]. These discrepancies seem to be a result of differences in filtration methods employed (use of 0.1 μm vs. 0.2 μm filters) in generating EV-depleted plasma. Relative contribution of RBCEVs to TG in healthy individuals is likely lower than circulating EVs generated by platelets, endothelial cells, and leukocytes [140].

Elevated levels of circulating procoagulant EVs and their contribution towards hypercoagulability and increased thrombosis of hemolytic disorders including SCA, beta thalassemia (BT), and paroxysmal nocturnal hemoglobinuria (PNH) are reported [142,143]. Of these hemolytic conditions, contribution of RBCEVs to the pathophysiology of SCA is particularly well studied. Presence of RBCEVs in SCA patient blood was first described in 1982 [144]. Since then, multiple studies identified elevated levels of RBCEVs in SCA patient plasma compared to healthy controls and suggested their role in the hypercoagulability of SCA [145,146,147,148,149,150,151]. Some studies identified further increases in the levels of RBCEVs in SCA patients during crisis compared to SCA patients at steady state [149,151,152,153]. However, complicating interpretation of data some studies have identified no significant differences in EV generation in SCA [145,146], while other experimental findings suggest increased levels of platelet-derived microparticles compared to RBCEVs in SCA [146,147,148,150,151,153,154,155]. Research efforts have attempted to assess RBCEVs in SCA patients to categorize non-severe and severe vaso-occlusive crises based on their circulating levels [150]. Further, comparison of circulating EVs between SCA and Hb SC (HbSC) genotype patients identified significantly higher levels of total microparticles including both RBC- and platelet-derived EVs in SC compared to HbSC patients [155].

In a study by Shet et al., addition of SCA-patient-derived circulatory EVs to plasma increased clotting time compared to control EVs from healthy control subjects [145]. Addition of an anti-tissue factor antibody partially inhibited the procoagulant activity in this study suggesting the presence of both tissue factor dependent and independent coagulation activity [145]. Similarly, in a study by van Beers et al., total TG correlated with the number of circulatory EVs and the thrombin activity was significantly blocked by anti-human factor XI, unaffected by anti-human factor VII, and modestly increased by anti-tissue factor pathway inhibitor antibody, upon co-incubation [146]. Importantly, the extent of factor XI inhibition correlated with the number of RBCEVs, suggesting the primary contribution of RBCEVs to the observed thrombogenicity. Additionally, the number of RBCEVs correlated with markers of hemolysis (Hb and lactate dehydrogenase), platelet/endothelial cell activation (vWF antigen), and fibrinolysis (prothrombin fragment F1+2 and D-dimers) analyzed in this study [146]. Gerotziafas et al. identified acceleration of the propagation phase of TG in SCA patient plasma and determined that RBCEVs expressing PS are the major contributory factors. Further, the authors demonstrate that hydroxyurea treatment reduced the number of procoagulant RBCEVs and TG in SCA patients [147]. In this context, it must be added that variable effects of hydroxyurea treatment on RBCEV production were demonstrated [[147,154]. In a study assessing the effects of red cell exchange on circulatory EVs accumulation, a significant decrease in RBC-derived, but not platelet-derived EVs was observed [148]. Overall, RBCEVs are reported to be elevated in SCA and potentially contribute to SCA pathophysiology and coagulopathy. Nonetheless, differences in SCA severity and progression of the disease in patients as well as experimental study design were identified as potential reasons for discrepancies in data across studies assessing RBCEVs role in SCA [156].

BT intermedia and major forms are both associated with a hypercoagulable state and increased incidence of thrombosis [157,158]. Elevated levels of circulating EVs including RBCEVs in BT are reported in multiple studies [159,160,161,162,163,164,165]. In splenectomized BT patients, a significant increase in the number of circulating RBCEVs is observed compared to non-splenectomized patients [159,160,164,165]. Despite elevated levels, contribution of RBCEVs to hypercoagulability of BT is not clear. In a study by Chaichompoo et al., EVs derived from platelets are suggested to be the primary pro-coagulant EVs in BT since the levels of microparticles with prothrombinase activity correlated with platelet numbers [162]. Tripodi et al. identified hypercoagulability in patients using whole blood thromboelastography, but not by TG in platelet-poor plasma, and concluded platelet and/or blood cell components as the primary determinants of thrombotic risk in BT [166].

PNH is a hematological disorder that is clinically associated with complement-driven intravascular hemolysis, thrombosis, and anemia [167,168]. Thromboembolism is identified as the most common cause of mortality in patients suffering from attacks of PNH and accounts for up to 67% of deaths in this rare disease [167,168]. During bouts of PNH, EVs are released from complement activated RBCs and demonstrate increased TG when compared to EVs released from normal RBCs. This may indicate a thrombogenic RBCEV subtype in certain patients that contributes toward hypercoagulability and thrombosis during bouts of PNH [169]. However, lower levels of RBC-derived procoagulant EVs are found when compared to platelet EV concentrations in PNH patient plasmas [170,171]. Overall, these findings suggest a lesser contribution of RBCEVs to the prothrombotic state observed in most PNH patients [167,168].

The contributory role of reticulocyte-derived EVs to coagulopathy is not clear from the existing literature. Generation of exosomes from reticulocytes is well established, but their ability to produce MVs requires a more extensive understanding. The membranes of reticulocyte-derived exosomes contain 20% exposed PS [172] and Mankelow et al. [173] reported elevated levels of reticulocytes expressing PS-exposed autophagic vesicles in SCD patients and proposed that failure to remove these vesicles by spleen could contribute to a hypercoagulable state and increased thrombotic events. Considering the primary contribution of PS to coagulation and immunomodulatory properties of RBCEVs [122,126,127], studies are needed to explore the contribution of reticulocyte-derived EVs to the pathophysiology of disease conditions, specifically hemolytic disorders where the numbers of reticulocytes in peripheral blood are elevated.

In summary, RBCEVs are elevated upon storage and in several genetic non-malignant hematologic disease states. Together with alterations in cellular components, pro-coagulant factors, fibrinolytic factors, and platelet-derived EVs, our understanding of RBCEVs role as contributors toward hypercoagulability and thrombogenicity continues to evolve.

## 5. Therapeutic Opportunities for RBCEVs

Beginning in 2010, EV-based therapeutics research has focused on several areas of disease including cancer [174,175], cardiovascular disease [176,177], central nervous system (CNS) disorders [178], as well as pulmonary [179], hepatic [180], and renal disease [181]. Delivery of miRNA or siRNA payload using EVs has focused on anti-cancer treatments in rodent models of glioma [182], carcinoma [183], and pancreatic cancer [184,185]. Data from these preliminary studies suggest that EVs effectively enter tissue parenchymal and tumor cells delivering their RNA cargo. When compared with liposomes, preliminary studies further suggest that EVs minimize immune response and may offer a novel delivery system [174,175,182].

Human RBCs can be stimulated to produce EVs for RNA therapies because they lack both nuclear and mitochondrial DNA [186]. A RBCEV platform was used to deliver RNA-based therapeutics to treat solid and liquid tumors in breast cancer and acute myeloid leukemia (AML) cell lines, respectively [187]. In these proof-of-concept studies RNA-loaded RBCEVs were absorbed by both breast cancer and AML cells with high efficiency. In AML, MOLM13 engrafted mice RBCEV-miR-125b antisense oligonucleotides suppressed miR-125b expression levels, cancer cell proliferation, and infiltration. Efficient engraftment of RBCEV-antisense oligonucleotides in human metastatic breast cancer MCF10CA1a cells were observed using in vivo fluorescent imaging. Lipophilic drugs such as camptothecin packaged within RBCEVs are observed to be taken up by lung carcinoma cells and show an improvement in targeted delivery in vivo when compared with synthetic lipid-based nanocarriers [188]. RBCEVs are effective packaging and delivery systems for iron oxide to target human bone marrow mesenchymal stem cells for magnetic resonance imaging studies [189]. However, the limitations associated with large-scale production and purification of natural exosomes were overcome by the production of exosome mimetics (EMs) from RBCs. Gangadaran et al. generated EMs from RBCs by a one-step extrusion method that had 130-fold greater yield compared to natural NVs generated from RBCs and displayed enhanced in vivo biodistribution [190].

Although small molecules or biologics remain the most common therapeutics, several limitations may apply including poor bioavailability, high dose requirements, non-specific targeting, drug resistance, and low therapeutic indices. These limitations have advanced with the advent of nanoparticle (NP)-based drug delivery formulations that display enhanced permeability and retention (EPR) effect, improved stability, favorable toxicological profiles, optimized biocompatibility, longer shelf lives, as well as increased therapeutic release efficiency. However, NPs often lack selective features for tissue targeting (e.g., selective tumor targeting/tumor cell binding) and extended blood circulation times, thus limiting their clinical applications to date [191]. This is in part due to immune clearance of NPs by the reticuloendothelial and mono-nuclear phagocytic systems. To overcome this issue, surface functionalization of NPs with polyethylene glycol (PEG) were incorporated into formulations to improve pharmacokinetic exposures by reducing rates of immune clearance [192]. However, appearance of anti-PEG immune response upon repeated administrations resulted in lowered drug efficacy, shifting the focus towards biomimetic particles that mimic self and therefore do not trigger an immune response [193]. Furthermore, biomimetic particles comprise several biological features of biocompatibility, biodegradability, selective tumor targeting, and extended circulatory half-life. The principle of generating biomimetic NPs involve “coating or camouflaging” NPs with membranes derived from bacteria, tumor cells, lymphocytes, platelets, leucocytes, and RBCs. Among these, RBCs represent an excellent source of membranes due to their abundance (5 billion RBC/mL of blood), biocompatibility, extended circulation (120-day life-span), and lack of internal organelles and nucleus easing the membrane extraction procedures [194,195,196,197,198,199].

Several integral membrane proteins in RBC membranes serve as self-markers that prevent immune clearance and allow extended circulation. For instance, CD47 serves as a “don’t eat me” signal which interacts and signals via signal regulatory protein alpha (SIRPα) on macrophage surface and thus inhibits macrophage engulfment of RBCs [200,201]. Similarly, membrane proteins, C8 binding protein (C8bp) [202], homologous restriction protein (HRP) [203], decay accelerating factor (DAF) [204], membrane cofactor protein (MCP), complement receptor 1 (CR1), and CD59 prevent attack by complement complexes [205]. Thus, surface proteins on RBC membranes present themselves as “self” facilitating extended circulation. Consistent with this, RBC membrane-coated NPs exhibited an elimination half-life of 39.6h, compared to PEGylated NPs (15.8h) [206].

Generation of RBC membrane-coated nanoparticles (NPs) involves the production of RBC membrane-derived vesicles (RVs) followed by vesicle-NP fusion [207]. RVs are generated in two steps. First, hypotonic treatment of pure RBCs separated from blood serum and buffy coat followed by a centrifugation step results in the removal of intracellular components and generates RBC ghost membranes. This step is followed by sonication and sequential extrusion through various pore size polycarbonate membranes to achieve target vesicle size [207,208,209]. Second, various methods of vesicle-nanoparticle fusion exist including co-extrusion, microfluidic electroporation and cell membrane templated polymerization. Both mechanical extrusion and microfluidic electroporation methods incorporate principles of interfacial interactions where negative charged sialyl residues on surface polysaccharides confer a charge asymmetry, which facilitates interactions between RVs and NPs [210]. Negatively charged sialyl moieties on the outer membrane side undergo a strong electrostatic repulsion with negatively charged NPs to fuse with the intracellular membrane side in a right-side-out-membrane orientation [211]. In contrast, negatively charged sialyl moieties likely display strong affinity towards positively charged NPs resulting in the collapse of lipid bilayer and hindering the local arrangement necessary for lipid covering [211].

In the co-extrusion method, NPs are fused with RVs via mechanical extrusion. Depending on NP size, NP and RV mixture is extruded through porous membranes of different sizes prior to water bath sonication. The mechanical force during extrusion allows for NPs passage through the lipid bilayer, causing vesicle-particle fusion [210]. After repeated extrusions, the excess RVs are removed by centrifugation, and RV-NP precipitates representing the final product are collected and dispersed for future use [209]. An example of the microfluidic electroporation process involves iron oxide (Fe_3_O_4_) magnetic nanoparticles (MNPs) and RVs combined on a microfluidic chip. The mixture is flown through an electroporation zone [211]. The electrical pulses can break down the dielectric layer on the cell membranes to create multiple transient pores and allow integration of MNPs into RVs [212]. Upon integration, RV-MNPs are collected from chips and used for in vivo performance tests. Under conditions where interfacial interactions between RV membranes and NPs are hindered due to the use of non-compliant NP core materials, cell membrane-template polymerization method are incorporated to synthesize polymer cores via in situ polymerization to form cell membrane-coated nanogels [213]. Several polymeric core NPs including poly(caprolactone) PCL NPs [214], poly pyrrole (PPy) NPs [215,216], poly (lactide acid) (PLA) NPs [217,218], poly (D, L-lactide-co-glycolide) (PLGA) NPs [219,220,221,222] and other types, including Fe_3_O_4_ MNPs [223,224,225], MNP clusters [226,227,228], mesoporous silica NPs [229,230], up conversion NPs (UCNPs) [231,232,233,234,235], gold nanoparticles (AuNPs) [230,236,237], and gelatin NPs [238,239] have been successfully coated with RBC membranes and evaluated for their potential use in several biomedical applications including chemotherapy, phototherapy, and diagnostic imaging [240]. For an in-depth review of preparation and therapeutic uses of RVs, readers are directed to Castro E. et al. [240].

## 6. Conclusions

EVs comprising exosomes and MVs originate from endosomes and PM, respectively. Although EVs in general are best known for their functions in intercellular communication, EV biogenesis and release have integral roles in RBC maturation, ageing and disease. MV release during RBC maturation and ageing involves disposal of unwanted membrane proteins and damaged Hb coupled with membrane remodeling. Furthermore, both in vitro storage and hematological diseases are known to generate MVs that contribute toward immunomodulation, inflammation, and coagulation.

Increased circulating EVs may contribute toward hypercoagulability and thrombosis after refrigerator storage and are suggested to interfere with nitric oxide signaling, which leads to endothelial dysfunction as well as perfusion and oxygenation deficiency. Hemolytic disorders including SCA, BT, and PNH are a primary focus of this short review and these disease states continue to be the most studied as they relate to the biogenesis and pathophysiology of RBCEVs. However, there remain critical questions that apply to the true impact of RBCEVs generated under storage conditions or during disease. While there is some indication as to the role of RBCEVs under certain circumstances, the literature remains largely inconsistent in its results and conclusions. Nonetheless, therapeutics based on RBCEVs are being studied with some degree of sophistication. This is largely inconsistent with the proof-of-concept required for therapeutics development and completely void of the potential for toxicological risk assessment.

Based on our assessments of the literature to date, it remains clear that a greater understanding of RBCEV generation, physiology, and pathophysiology should be a goal of future scientific endeavors. More specifically, studies suggest that RBCEVs play relevant communicative roles that alter tissue adaptation to disease and environment. This work represents a logical and intriguing role for RBCs given their abundance and proximity to tissues and cells. Nonetheless, future studies require more in-depth assessments of how communication between RBCs and tissues occurs. These studies should be directed at the origin, packaging, release triggers, and target tissue phenotype altering effects specific to RBC miRNA transfer and the true relevance of such processes.

## Supplementary Materials

The following are available online at https://www.mdpi.com/1422-0067/22/1/153/s1, Table S1: Selective studies evaluating the implications of RBC extracellular vesicles: Due to overlapping data selective studies on RBCEVs are provided in chronological order with emphasis on RBC source, composition, size and key findings.

## Abbreviations

AChE	Acetylcholinesterase
AFM	Atomic force microscopy
AML	Acute myeloid leukemia
ARRDC	Arrestin-domain-containing protein 1
ARMMs	Arrestin-domain-containing pro­tein 1 (ARRDC1) -mediated microvesicles
ATP	Adenosine triphosphate
BT	Beta thalassemia
CNS	Central nervous system
DLS	Dynamic light scattering
DPG	2,3-diphosphoglycerate
EM	Electron microscopy
EM	Exosome mimetics
ESCRT	Endosomal sorting complex required for transport
ETP	Endogenous thrombin potential
EVs	Extracellular vesicles
FC	Flow-cytometry
GPI	Glycosylphosphatidylinositol
Hb	Hemoglobin
HbSC	Hemoglobin SC
HRP II	Plasmodium falciparum histidine rich protein II
Ig	Immunoglobulins
ILVs	Intra-luminal vesicles
ITP	Immune thrombocytopenia
LC-MS	Tandem mass spectrometry
LPA	lysophosphatidic acid
miRNA	MicroRNA
MNP	Magnetic nanoparticles
MVs	Microvesicles
MVB	Multivesicular bodies
NO	Nitric oxide
NP	Nanoparticles
NTA	Nano-tracker analysis
PBMCs	Peripheral blood mononuclear cells
PC	Phosphatidylcholine
PCA-ELISA	Perchloric acid- enzyme linked immunosorbent assay
PE	Phosphatidylethanolamine
PG	Phosphatidylglycerol
PI	Phosphoinositides
PM	Plasma Membrane
PMA	Phorbol-12-myristate-13-acetate
PNH	Paroxysmal nocturnal hemoglobinuria
pRBCs	Plasmodium falciparum red blood cells
PS	Phosphatidylserine
RBCs	Red blood cells
ROS	Reactive oxygen species
RV	RBC membrane-derived vesicles
SCD	Sickle cell disease
SM	Sphingomyelin
S1P	Sphingosine 1-phosphate
T2MD	Type 2 diabetes
TEM	Transmission electron microscope
TG	Thrombin generation
TI	Thalassemia intermedia
TF	Tissue factor
TLC	Thin-layer chromatography
TRPS	Tunable Resistive Pulse Sensing
UC	Ultracentrifugation
