# Supplementary material for: Extracellular Vesicles from Red Blood Cells and Their Evolving Roles in Health, Coagulopathy and Therapy
Source: Int J Mol Sci. 2020 Dec 25;22(1):153. doi: 10.3390/ijms22010153 (PMC7796437; doi:10.3390/ijms22010153)
Supplement: Supplementary file 1 [file ijms-22-00153-s001.pdf]

International Journal of
Molecular Sciences

MDPI

Supplementary information. Table S1

Study	Techniques/Composition/Size	Key findings
[1]	Techniques: EM and Western blotting Composition: band 3, aggregated hemoglobin, and lipid raft proteins were present in vesicles. Fas, FADD, procaspases 3 and 8, caspase 8 and caspase 3 cleavage products were present in vesicles after the 10th day, CD47 (after the 17th day), and immunoglobulin G.	During RBC storage, vesiculation enables RBCs to shed altered or harmful material.
[2]	Techniques: gel electrophoresis and semi-quantitative proteomic analysis, liquid chromatography, tandem mass spectrometry, Nano–LC-MS/MS Composition: Proteins: structural and transport proteins like Spectrin, ankyrin, Band 3, glucose transporter, protein 4.1, Protein 4.2, Protein 4.9, actin. Complement components, immunoglobulin, membrane and cytoskeletal proteins, Hb, metabolic enzymes, chaperone, proteasome, Small G protein, raft, nucleic acids, proteins associated with Oxidation, Signal transduction were present	Storage related changes in RBC membrane is attributed to cellular aging and vesiculation. Vesiculation helps in removing damaged membrane patches.
[3]	Techniques: AFM, detergent-resistant membrane analysis for raft protein content Composition: Acetylcholinesterase, band 3, CD55, stomatin, flotillin-1,2, and Duffy antigens.	Concluded that stomatin specific, raft-based process in storage associated RBC vesiculation.
[4]	Techniques: Qualitative and quantitative experiments using FC and proteomic techniques Composition: CD235a, stomatin (RBC band 7 integral membrane protein), carbonic anhydrases, 14-3-3 proteins, , Hb α, β subunits etc., see Table 1[4]	A 20-fold increase in RBCEVs after 50 days of storage at 4°C. Rhesus blood group antigens were present on EVs from stored RBCs.
[5]	-	RBCEVs are elevated in splenectomized ITP patients. Levels of RBCEVs correlated with elevated clotting factor activities (FVIII, FIX and FXI) and shortened aPTT. RBCEVs may contribute to increased risk of thrombosis and cardiovascular disease post-splenectomy.
[6]	Techniques: FC and thrombin generation assay. Composition: glycophorin A+	Circulatory levels of RBCEVs correlated with plasma levels of markers of hemolysis, platelet/endothelial cell activation, fibrinolysis and coagulation activation. procoagulant state in SCD patients is partially explained by the procoagulant effects of RBCEVs
[7]	Techniques: FC	RBCEVs scavenge nitric oxide at slightly less rate than cell free Hb but faster than RBC-encapsulated Hb.
[8]	Techniques: ZYMUPHEN MP-Activity Assay kit, FC	Procoagulant properties of RBCEVs from PNH patients could contribute to the thrombogenesis of PNH.

[9]	Techniques: FC	Thrombospondin-1 mediated RBCEV shedding and its involvement in acute vaso-occlusive events in Murine model of sickle cell disease.
[10]	Techniques: FC and procoagulant activity, TF, Thrombin generation, and FXIIa activity measurements.<br>Composition: glycophorin A and CD239	Blood from healthy individuals and the FXII-deficient donor, not using medication. RBCEVs alongside platelet EVs trigger thrombin generation via factor XIIa, independent of tissue factor.
[11]	Techniques: TEM, FC, Western blotting, AFM<br>Contents: negatively charged phospholipids	RBCEVs from stored blood induced thrombin generation in a FXI -dependent and tissue-factor-independent manner.
[12]	RBCEVs 106.5 ± 1.12 nm<br>*P.falciparum* infected RBCEVs ~120 nm	EVs secreted from *Plasmodium falciparum*-infected RBCs mediate communication between cells.
[11]	Techniques: UC, TEM, FC, Western blotting<br>Composition: negatively charged phospholipids, 97% of RBCEVs expressed erythroid marker CD235a	RBCEVs from stored blood induced thrombin generation in an FXI-dependent and tissue-factor-independent manner.
[13]	Techniques: UC, FC, Cryogenic TEM<br>Composition: CD235a	Under in vitro conditions, hemoglobin containing RBCEVs bound and transferred heme to human umbilical cord vascular endothelial cells and induced oxidative stress and apoptosis. Injection of hemoglobin containing RBCEVs injected in sickle cell disease mouse model resulted in rapid vaso-occlusions in kidneys.
[14]	Techniques: FC, Acetylcholinesterase activity assay<br>Composition: No change in glycosylphosphatidylinositol (GPI)-linked enzyme CD59 and acetylcholinesterase protein levels, decrease in glycophorin A levels	In vitro generated vesicles show loss in AChE activity, but not of AChE expression and AChE could be used as a measure of RBC ageing in vivo.
[15]	Techniques: TRPS, FC, DLS	Length of storage impacts the EV size and concentration in RBC products. TRPS: < 200 nm sized EVs increased throughout storage. TRPS and FC: ≥ 200 nm sized EVs increased in RBC concentrates by day 42/43 compared to EVs present on day 3. DLS: only an increase in the zeta-average size was observed during storage.
[16]	Techniques: NTA, mass spectrometric and chromatographic techniques Composition: CD235a and Hb, PC-diacyl species, PE-alkenyl-acyl and diacyl species, SM and PS. 90% of particles 100–300 nm in size	Absence of raft lipids in stored RBCEVs suggest that the vesiculation process does not involve raft-based mechanism. Moreover, phospholipid composition of red blood cells and corresponding extracellular vesicles were similar.
[17]	Techniques: Western blotting, TLC, AFM, NTA, mass spectrometric analysis. Composition: Hb, band 3, protein 4.1 and 4.2, spectrin, stomatin. Mean size of the vesicle's AFM: 88 ± 2 nm; 94 ± 5 nm; NTA 71 ± 1 nm.	Calcium stimulated RBCEVs from healthy individuals and hereditary spherocytosis patient derived RBCEVs have altered protein composition. Healthy individual EVs resemble fluid liposomes and those from hereditary spherocytosis patients have a softer membrane, which might be responsible for increased rate of EV budding seen in HS patients.
[18]	Techniques: FC, TRPS Assay. Composition: ATP, 2,3- DPG levels saw a significant decrease.	Length of storage and blood manufacturing protocol determine the size and concentration of the EVs.

[19]	Techniques: TEM and NTA<br>Composition: Abundance of human miRNAs and tRNA-derived fragments. Y-RNAs, vault RNAs, snoRNAs and piRNAs were also present. 305 Human regulatory miRNAs were identified in at least one sample and 61 miRNAs were present in all the 3 samples. miR451a miR486-5p (22.51%), miR92a-3p (9.37%) and miR103a-3p (6.34%) were abundant. EVs enriched in stomatin and RESA and negative for the endoplasmic reticulum Bip	EVs derived from in vitro cultures of *P. falciparum* and human iRBCs. Plasmodial extracellular RNAs are associated with EVs and participate in RNA cargo delivery to human endothelial cells.
[20]	-	RBCEVs positively correlated with fasting blood glucose and predicted the presence of T2DM independent of platelet derived vesicles.
[21]	Techniques: FC, PCA ELISA. Composition: Total EVs: Majority of RBC origin - 87% positive for Glycophorin A and 44% for annexin V	A 17.6-fold increase in RBCEVs after 6 weeks of storage. RBCEVs are procoagulant and their reduction can minimize transfusion-induced thrombotic complications.
[22]	Techniques: TEM, western blotting, and DLS. MicroRNA microarray, qRT-PCR, bioinformatics analysis<br>Composition: Short RNA are abundant compared to long RNA. Size of RBCEVs were 64.08 nm	Seventy-eight miRNAs detected in three exosome samples. Top 3 abundant miRs: miR-125b-5p, miR-4454, and miR-451a. miR-4454 and miR-451a increased with increased storage time
[23]	Techniques: FC<br>Composition: PNH patients lacked PS exposure on RBCEVs	No significant difference between RBCEV concentration between PNH patients and controls. CD59-high RBCEV was more in control plasma donors compared to PNH patients.
[24]	Techniques: FC, TEM<br>Composition: miRNA-150, CD9 and CD81	EVs generated by the intravenous administration of syngeneic RBCs suppress delayed-type hypersensitivity reactions through miRNA-150 dependent mechanisms.
[25]	Techniques: FC, NTA, Western blotting, size exclusion chromatography, thrombin generation assay.<br>Composition: tetraspanin CD63 (low abundance). α-Actinin1(abundance), Alix (barely detected)	RBCEVs induce human coagulation factor XII dependent thrombin generation in the absence of corn trypsin inhibitor
[26]	Techniques: FC, NTA, TEM, Lipidomic analysis, Cell targeting analysis.<br>Composition: parasite protein HRP II and membrane protein Glycophorin A.<br>Size of RBCEVs 211.0 ± 8.1 nm; were slightly larger than pRBCEVs 179.7 ± 6.8 nm;	No significant qualitative differences were observed between EVs from *Plasmodium falciparum*-infected RBCs (pRBCs) and normal RBCs.<br>pRBCEVs loaded with antimalarial drugs atovaquone and tafenoquine inhibited in vitro *P. falciparum* growth more efficiently compared to non-encapsulated drugs. The pRBCEVs have an impact on the efficacy of hydrophobic drugs to treat malaria.
[27]	Techniques: Scanning Electron Microscopy, Spectrin Immunofluorescence,<br>Composition: EVs from stored blood in K+/EDTA tubes for up to 4 weeks at 4°C - enrichment of ceramide and dihydroceramide species at the end of the storage period. SM and PC decreased upon storage. Lyso-PI and	Four successive events are involved in RBCEV shedding from lipid domains: 1. cholesterol domain decrease, 2. oxidative stress, 3. sphingomyelin/sphingomyelinase/ceramide/calcium alteration, and 4. phosphatidylserine exposure.

	Lysyl-PG increased between the 3rd and 4th weeks Lyso-PC and Lyso-PE species remained almost stable.	
[28]	Techniques: FC, cell-flow-properties-analyzer Composition: CD235, CD35, and Annexin V	EV formation plays a vital role in increased RBC rigidity during storage
[29]	Techniques: DLS and NTA<br>Composition: PS	FXII and prekallikrein pathways are activated by RBCEV (larger microvesicles) leading to FIX activation by the classic FXIIa-FXI-FIX pathway and direct kallikrein pathway. These pathways are potential targets to prevent thrombotic and/or inflammatory complications of red blood cell transfusion.
[30]	Techniques: Cryo-TEM, proteomic analysis - LC-MS/MS, metabolomics, FC<br>Composition: RBCEVs enriched in cytoplasmic protein hemoglobin, the membrane protein band 3, the plasma-derived IgG, stomatin, flotillin-1 and flotillin-2	There was change in EV composition throughout storage confirmed by morphological and metabolic analysis.<br>For a functional and intact RBCs membrane lipid and protein compartments interaction is necessary.

a. **TableS1:** Selective studies evaluating the implications of RBC extracellular vesicles: Due to overlapping data selective studies on RBCEVs are provided in chronological order with emphasis on RBC source, composition, size and key findings. Abbreviations: AChE- Acetylcholinesterase, AFM- Atomic force microscopy, ATP- Adenosine triphosphate, DLS- dynamic light scattering, DPG- 2,3-diphosphoglycerate, EM- Electron microscopy, FC- Flow-cytometry, GPI- glycosylphosphatidylinositol, Hb- Hemoglobin, HRP II- Plasmodium falciparum histidine rich protein 2, ITP- Immune thrombocytopenia, LC-MS/MS- Tandem mass spectrometry, miRNA- microRNA, NTA- Nano-tracker analysis, PCA-ELISA- perchloric acid enzyme-linked immunosorbent assay, PC- phosphatidylcholine, PE- phosphatidylethanolamine, PG- phosphatidylglycerol, PI- phosphoinositides, PNH- Paroxysmal nocturnal hemoglobinuria pRBCs- *Plasmodium falciparum* red blood cells, PS- Phosphatidylserine, ROS- reactive oxygen species SCD- Sickle cell disease, SM- Sphingomyelin, T2DM- Type 2 diabetes, TEM- transmission electron microscopy, TF- tissue factor, TLC- Thin-layer chromatography, TRPS- Tunable Resistive Pulse Sensing, UC- Ultracentrifugation,

References

1. Kriebardis, A.G.; Antonelou, M.H.; Stamoulis, K.E.; Economou-Petersen, E.; Margaritis, L.H.; Papassideri, I.S. RBC-derived vesicles during storage: ultrastructure, protein composition, oxidation, and signaling components. *Transfusion* **2008**, *48*, 1943-1953, doi:10.1111/j.1537-2995.2008.01794.x.
2. Bosman, G.J.C.G.M.; G., G.J.C.; Lasonder, E.; Luten, M.; Roerdinkholder-Stoelwinder, B.; Novotný, V.M.J.; Bos, H.; De Grip, W.J. The proteome of red cell membranes and vesicles during storage in blood bank conditions. *Transfusion* **2008**, *48*, 827-835, doi:10.1111/j.1537-2995.2007.01630.x-i2.
3. Salzer, U.; Zhu, R.; Luten, M.; Isobe, H.; Pastushenko, V.; Perkmann, T.; Hinterdorfer, P.; G., G.J.C. Vesicles generated during storage of red cells are rich in the lipid raft marker stomatin. *Transfusion* **2008**, *48*, 451-462, doi:10.1111/j.1537-2995.2007.01549.x.
4. Rubin, O.; Crettaz, D.; Canellini, G.; Tissot, J.D.; Lion, N. Microparticles in stored red blood cells: an approach using flow cytometry and proteomic tools. *Vox Sang.* **2008**, *95*, 288-297, doi:10.1111/j.1423-0410.2008.01101.x.
5. Fontana, V.; Jy, W.; Ahn, E.R.; Dudkiewicz, P.; Horstman, L.L.; Duncan, R.; Ahn, Y.S. Increased procoagulant cell-derived microparticles (C-MP) in splenectomized patients with ITP. *Thromb. Res.* **2008**, *122*, 599-603, doi:10.1016/j.thromres.2007.12.022.
6. van Beers, E.J.; Schaap, M.C.L.; Berckmans, R.J.; Nieuwland, R.; Sturk, A.; van Doormaal, F.F.; Meijers, J.C.M.; Biemond, B.J.; group, C.s. Circulating erythrocyte-derived microparticles are associated with coagulation activation in sickle cell disease. *Haematologica* **2009**, *94*, 1513-1519, doi:10.3324/haematol.2009.008938.
7. Donadee, C.; Raat, N.J.H.; Kanias, T.; Tejero, J.; Lee, J.S.; Kelley, E.E.; Zhao, X.; Liu, C.; Reynolds, H.; Azarov, I., et al. Nitric oxide scavenging by red blood cell microparticles and cell-free hemoglobin as a mechanism for the red cell storage lesion. *Circulation* **2011**, *124*, 465-476, doi:10.1161/CIRCULATIONAHA.110.008698.
8. Kozuma, Y.; Sawahata, Y.; Takei, Y.; Chiba, S.; Ninomiya, H. Procoagulant properties of microparticles released from red blood cells in paroxysmal nocturnal haemoglobinuria. *Br. J. Haematol.* **2011**, *152*, 631-639, doi:10.1111/j.1365-2141.2010.08505.x.
9. Camus, S.M.; Gausserès, B.; Bonnin, P.; Loufrani, L.; Grimaud, L.; Charue, D.; De Moraes, J.A.; Renard, J.-M.; Tedgui, A.; Boulanger, C.M., et al. Erythrocyte microparticles can induce kidney vaso-occlusions in a murine model of sickle cell disease. *Blood* **2012**, *120*, 5050-5058, doi:10.1182/blood-2012-02-413138.

10. Van Der Meijden, P.E.J.; Van Schilfgaarde, M.; Van Oerle, R.; Renné, T.; ten Cate, H.; Spronk, H.M.H. Platelet- and erythrocyte-derived microparticles trigger thrombin generation via factor XIIa. *J. Thromb. Haemost.* **2012**, *10*, 1355-1362, doi:10.1111/j.1538-7836.2012.04758.x.
11. Rubin, O.; Delobel, J.; Prudent, M.; Lion, N.; Kohl, K.; Tucker, E.I.; Tissot, J.-D.; Angelillo-Scherrer, A. Red blood cell-derived microparticles isolated from blood units initiate and propagate thrombin generation. *Transfusion* **2013**, *53*, 1744-1754, doi:10.1111/trf.12008.
12. Regev-Rudzki, N.; Wilson, D.W.; Carvalho, T.G.; Sisquella, X.; Coleman, B.M.; Rug, M.; Bursac, D.; Angrisano, F.; Gee, M.; Hill, A.F., et al. Cell-cell communication between malaria-infected red blood cells via exosome-like vesicles. *Cell* **2013**, *153*, 1120-1133, doi:10.1016/j.cell.2013.04.029.
13. Camus, S.M.; De Moraes, J.A.; Bonnin, P.; Abbyad, P.; Le Jeune, S.; Lionnet, F.; Loufrani, L.; Grimaud, L.; Lambry, J.-C.; Charue, D., et al. Circulating cell membrane microparticles transfer heme to endothelial cells and trigger vasoocclusions in sickle cell disease. *Blood* **2015**, *125*, 3805-3814, doi:10.1182/blood-2014-07-589283.
14. Freitas Leal, J.K.; Adjobo-Hermans, M.J.W.; Brock, R.; Bosman, G.J.C.G.M. Acetylcholinesterase provides new insights into red blood cell ageing in vivo and in vitro. *Blood Transfus.* **2017**, *15*, 232-238, doi:10.2450/2017.0370-16.
15. Almizraq, R.J.; Seghatchian, J.; Holovati, J.L.; Acker, J.P. Extracellular vesicle characteristics in stored red blood cell concentrates are influenced by the method of detection. *Transfus. Apher. Sci.* **2017**, *56*, 254-260, doi:10.1016/j.transci.2017.03.007.
16. Laurén, E.; Tigistu-Sahle, F.; Valkonen, S.; Westberg, M.; Valkeajärvi, A.; Eronen, J.; Siljander, P.; Pettilä, V.; Käkelä, R.; Laitinen, S., et al. Phospholipid composition of packed red blood cells and that of extracellular vesicles show a high resemblance and stability during storage. *Biochim. Biophys. Acta Mol. Cell Biol. Lipids* **2018**, *1863*, 1-8, doi:10.1016/j.bbalip.2017.09.012.
17. Vorselen, D.; van Dommelen, S.M.; Sorkin, R.; Piontek, M.C.; Schiller, J.; Döpp, S.T.; Kooijmans, S.A.A.; van Oirschot, B.A.; Versluijs, B.A.; Bierings, M.B., et al. The fluid membrane determines mechanics of erythrocyte extracellular vesicles and is softened in hereditary spherocytosis. *Nat. Commun.* **2018**, *9*, 4960, doi:10.1038/s41467-018-07445-x.
18. Almizraq, R.J.; Holovati, J.L.; Acker, J.P. Characteristics of Extracellular Vesicles in Red Blood Concentrates Change with Storage Time and Blood Manufacturing Method. *Transfus. Med. Hemother.* **2018**, *45*, 185-193, doi:10.1159/000486137.
19. Babatunde, K.A.; Mbagwu, S.; Hernández-Castañeda, M.A.; Adapa, S.R.; Walch, M.; Filgueira, L.; Falquet, L.; Jiang, R.H.Y.; Ghiran, I.; Mantel, P.-Y. Malaria infected red blood cells release small regulatory RNAs through extracellular vesicles. *Sci. Rep.* **2018**, *8*, 884, doi:10.1038/s41598-018-19149-9.
20. Gkaliagkousi, E.; Nikolaidou, B.; Gavriilaki, E.; Lazaridis, A.; Yiannaki, E.; Anyfanti, P.; Zografou, I.; Markala, D.; Douma, S. Increased erythrocyte- and platelet-derived microvesicles in newly diagnosed type 2 diabetes mellitus. *Diab. Vasc. Dis. Res.* **2019**, *16*, 458-465, doi:10.1177/1479164119844691.
21. Hashemi Tayer, A.; Amirizadeh, N.; Ahmadinejad, M.; Nikougoftar, M.; Deyhim, M.R.; Zolfaghari, S. Procoagulant Activity of Red Blood Cell-Derived Microvesicles during Red Cell Storage. *Transfus. Med. Hemother.* **2019**, *46*, 224-230, doi:10.1159/000494367.
22. Huang, H.; Zhu, J.; Fan, L.; Lin, Q.; Fu, D.; Wei, B.; Wei, S. MicroRNA Profiling of Exosomes Derived from Red Blood Cell Units: Implications in Transfusion-Related Immunomodulation. *Biomed Res. Int.* **2019**, *2019*, 2045915, doi:10.1155/2019/2045915.
23. Freitas Leal, J.K.; Preijers, F.; Brock, R.; Adjobo-Hermans, M.; Bosman, G. Red Blood Cell Homeostasis and Altered Vesicle Formation in Patients With Paroxysmal Nocturnal Hemoglobinuria. *Front. Physiol.* **2019**, *10*, 578, doi:10.3389/fphys.2019.00578.
24. Nazimek, K.; Bustos-Morán, E.; Blas-Rus, N.; Nowak, B.; Ptak, W.; Askenase, P.W.; Sánchez-Madrid, F.; Bryniarski, K. Syngeneic red blood cell–induced extracellular vesicles suppress delayed-type hypersensitivity to self-antigens in mice. *Clinical & Experimental Allergy* **2019**, *49*, 1487-1499, doi:10.1111/cea.13475.
25. Tripisciano, C.; Weiss, R.; Karuthedom George, S.; Fischer, M.B.; Weber, V. Extracellular Vesicles Derived From Platelets, Red Blood Cells, and Monocyte-Like Cells Differ Regarding Their Ability to Induce Factor XII-Dependent Thrombin Generation. *Front Cell Dev Biol* **2020**, *8*, 298, doi:10.3389/fcell.2020.00298.
26. Borgheti-Cardoso, L.N.; Kooijmans, S.A.A.; Chamorro, L.G.; Biosca, A.; Lantero, E.; Ramírez, M.; Avalos-Padilla, Y.; Crespo, I.; Fernández, I.; Fernandez-Becerra, C., et al. Extracellular vesicles derived from Plasmodium-infected and non-infected red blood cells as targeted drug delivery vehicles. *Int. J. Pharm.* **2020**, *587*, 119627, doi:10.1016/j.ijpharm.2020.119627.
27. Cloos, A.-S.; Ghodsi, M.; Stommen, A.; Vanderroost, J.; Dauguet, N.; Pollet, H.; D'Auria, L.; Mignolet, E.; Larondelle, Y.; Terrasi, R., et al. Interplay Between Plasma Membrane Lipid Alteration, Oxidative Stress and Calcium-Based Mechanism for Extracellular Vesicle Biogenesis From Erythrocytes During Blood Storage. *Front. Physiol.* **2020**, *11*, 712, doi:10.3389/fphys.2020.00712.
28. McVey, M.J.; Kuebler, W.M.; Orbach, A.; Arbell, D.; Zelig, O.; Barshtein, G.; Yedgar, S. Reduced deformability of stored red blood cells is associated with generation of extracellular vesicles. *Transfus. Apher. Sci.* **2020**, 10.1016/j.transci.2020.102851, 102851, doi:10.1016/j.transci.2020.102851.
29. Noubouossie, D.F.; Henderson, M.W.; Mooberry, M.; Ilich, A.; Ellsworth, P.; Piegore, M.; Skinner, S.C.; Pawlinski, R.; Welsby, I.; Renné, T., et al. Red blood cell microvesicles activate the contact system, leading to factor IX activation via 2 independent pathways. *Blood* **2020**, *135*, 755-765, doi:10.1182/blood.2019001643.
30. Freitas Leal, J.K.; Lasonder, E.; Sharma, V.; Schiller, J.; Fanelli, G.; Rinalducci, S.; Brock, R.; Bosman, G. Vesiculation of Red Blood Cells in the Blood Bank: A Multi-Omics Approach towards Identification of Causes and Consequences. *Proteomes* **2020**, *8*, doi:10.3390/proteomes8020006.
